# The Risk of Cholesteatoma in Individuals With First-degree Relatives Surgically Treated for the Disease

**DOI:** 10.1001/jamaoto.2023.0048

**Published:** 2023-03-16

**Authors:** Åsa Bonnard, Cecilia Engmér Berglin, Josephine Wincent, Per Olof Eriksson, Eva Westman, Maria Feychting, Hanna Mogensen

**Affiliations:** 1 Department of Clinical Sciences, Intervention and Technology, Karolinska Institutet, Stockholm, Sweden; 2 Medical Unit of ENT, Hearing and Balance, Karolinska University Hospital, Stockholm, Sweden; 3 Unit of Epidemiology, Institute of Environmental Medicine, Karolinska Institutet, Stockholm, Sweden; 4 Department of Molecular Medicine and Surgery, Karolinska Institutet, Stockholm, Sweden; 5 Department of Clinical Genetics, Karolinska University Hospital, Stockholm, Sweden; 6 Department of Surgical Sciences, Otorhinolaryngology, Uppsala University Hospital, Uppsala, Sweden; 7 Department of Clinical Sciences, Otorhinolaryngology, Umeå University, site Sundsvall, Umeå, Sweden

## Abstract

**Importance:**

Cholesteatoma in the middle ear is not regarded as a hereditary disease, but case reports of familial clustering exist in the literature, as well as observed familial cases in the clinical work. However, the knowledge regarding cholesteatoma as a hereditary disease is lacking in the literature.

**Objective:**

To assess the risk of cholesteatoma in individuals with a first-degree relative surgically treated for the same disease.

**Design, Setting, and Participants:**

In this nested case-control study in the Swedish population between 1987 and 2018 of first-time cholesteatoma surgery identified from the Swedish National Patient Register, 2 controls per case were randomly selected from the population register through incidence density sampling, and all first-degree relatives for cases and controls were identified. Data were received in April 2022, and analyses were conducted between April and September 2022.

**Exposure:**

Cholesteatoma surgery in a first-degree relative.

**Main Outcomes and Measures:**

The main outcome was first-time cholesteatoma surgery. The association between having a first-degree relative with cholesteatoma and the risk of cholesteatoma surgery in the index persons was estimated by odds ratios (ORs) and 95% CIs through conditional logistic regression analysis.

**Results:**

Between 1987 and 2018, 10 618 individuals with a first-time cholesteatoma surgery (mean [SD] age at surgery, 35.6 [21.5] years; 6302 [59.4%] men) were identified in the Swedish National Patient Register. The risk of having a cholesteatoma surgery was almost 4 times higher in individuals having a first-degree relative surgically treated for the disease (OR, 3.9; 95% CI, 3.1-4.8), but few cases were exposed overall. Among the 10 105 cases with at least 1 control included in the main analysis, 227 (2.2%) had at least 1 first-degree relative treated for cholesteatoma, while the corresponding numbers for controls were 118 of 19 553 control patients (0.6%). The association was stronger for individuals under the age of 20 years at first surgery (OR, 5.2; 95% CI, 3.6-7.6) and for a surgery involving the atticus and/or mastoid region (OR, 4.8; 95% CI, 3.4-6.2). There was no difference in the prevalence of having a partner with cholesteatoma between cases and controls (10 cases [0.3%] and 16 controls [0.3%]; OR, 0.92; 95% CI, 0.41-2.05), which implies that increased awareness does not explain the association.

**Conclusions and Relevance:**

In this Swedish case-control study using nationwide register data with high coverage and completeness, the findings suggest that the risk of cholesteatoma in the middle ear is strongly associated with a family history of the condition. Family history was nevertheless quite rare and can therefore only explain a limited number of all cases; these families could be an important source for information regarding the genetic background for cholesteatoma disease.

## Key Points

**Question:**

Is cholesteatoma in a first-degree relative associated with an increased risk of the disease?

**Findings:**

In this nationwide case-control study of first-time cholesteatoma surgeries including 10 618 cases and 21 235 controls in Sweden, the risk of cholesteatoma surgery for individuals with a first-degree relative treated for the disease was almost 4 times increased, but few cases were exposed.

**Meaning:**

This study suggests that a strong hereditary component in cholesteatoma disease exists, but this only explains a limited number of cholesteatoma cases.

## Teaser

This case-control study assesses the risk of cholesteatoma in individuals with a first-degree relative surgically treated for the same disease.

## Introduction

Cholesteatoma in the middle ear is an uncommon disease accompanied by risks for severe complications if not treated. The incidence of cholesteatoma is 6 to 9 per 100 000 inhabitants in developed countries and tends to be higher in children.^1,2,3^ A decline in incidence has been described over the last decades in several countries.^1,2,3^ There is a male dominance in having had cholesteatoma surgery.^4^

A cholesteatoma is typically defined as an acquired retraction pocket in the tympanic membrane that loses its ability to self-clean and thus starts to accumulate keratin debris.^5,6^ With time, the cholesteatoma grows and affects surrounding bone and other nerve and soft tissues, which can lead to hearing loss and disturbances of taste, balance, and facial nerve function. Infection of the cholesteatoma is common and seems to increase the rate of bone resorption.^7^ Cholesteatoma, if not treated, can lead to sinus thrombosis, meningitis, and intracranial abscess.

Several other types of cholesteatoma exists, including (1) acquired nonretraction pocket cholesteatoma emerging from a perforated tympanic membrane, (2) congenital cholesteatoma under an intact membrane, and (3) postsurgical cholesteatoma divided in recurrent or residual cholesteatoma.^6^ The treatment of the disease is surgical, but occasionally individuals will not undergo surgery, mostly due to medical reasons.

In the literature, cholesteatoma is in general not presented as a hereditary disease, but in clinical practice, histories of familial cases are accumulating. The results of a search in the literature are sparse, and in a 2018 review article by Jennings et al,^8^ only 35 articles were found on the subject, ranging from case reports of siblings to genetic testing. However, in 2009, Prinsley^9^ reported on family clustering of cholesteatoma in 12 families in the United Kingdom, the largest cohort yet reported in the literature. In 2019, a preliminary communication from the same research group^10^ showed the results of a genetic study of one of these families with the identification of 2 genes of interest.

To our knowledge, no previous population-based studies have examined heritability of cholesteatoma in a large scale. In Sweden, medical care is tax-funded and available for all citizens. Moreover, medical procedures are recorded in nationwide health registers that can be linked to other population registers using the unique personal identity number that is assigned to all residents in Sweden.^11^ This infrastructure enables the possibility for nationwide studies of rare diseases. In this study, the aim was to investigate the risk of cholesteatoma in individuals with a first-degree relative surgically treated for the same disease.

## Methods

### Study Design

This is a nationwide population-based case-control study nested within the Swedish total population between 1987 and 2018. The study was conducted by record-linkage of several Swedish national health data and population registers. The study has been approved by The Swedish Ethical Review Authority (2019-05190, 2020-000245, 2021-05727-02), and informed consent was waived due to the large sample size and the use of register data. The study follows the Strengthening the Reporting of Observational Studies in Epidemiology (STROBE) reporting guideline.

The rating of the quality of evidence was 3 due to the case-control study design. However, the study includes national, high quality register data covering a whole nation during a 30-year time period.

### Cases and Controls

Cases of cholesteatoma were identified from the Swedish National Patient Register.^12^ This register includes discharge diagnoses, as well as information on procedures performed, from all inpatient and specialized outpatient care. The coverage of inpatient care has been nationwide since 1987, and outpatient visits to specialized care (physicians) have been included since 2001.^12^ The register has in general a high sensitivity in regard to surgical interventions.^12^ All individuals with a diagnosis of cholesteatoma treated with cholesteatoma surgery between 1987 and 2018 (see eTable in the Supplement for diagnostic codes according to *International Classification of Diseases, Ninth Revision [ICD-9] *and* International Statistical Classification of Diseases and Related Health Problems, Tenth Revision [ICD-10]* and Swedish procedural codes) were defined as cases of cholesteatoma and included at the time of first-registered cholesteatoma surgery during the study period. Cases were further divided according to surgical approach of the cholesteatoma removal into atticus or mastoid, and other locations, as indicated in the registered procedural codes (eTable in the Supplement).

Through incidence density sampling, 2 controls per case were randomly selected from the general population using the Total Population Register^13^ and were matched to cases by age, sex, and municipality of residence at the date of surgery of the case (hereinafter, index date). The control selection was conducted by Statistics Sweden.

### Exposure

The exposure was defined as having a first-degree relative surgically treated for cholesteatoma. For cases and controls, all first-degree relatives (ie, biological parents, full and half siblings, and children) were identified using the Multi-generation Register^14^ (Figure). This national register enables linkage between children and parents for persons born in 1932 and later.^14^ In the next step, all first-degree relatives that were registered in Sweden any time between 1987 and 2018 were linked to the National Patient Register to assess if they had been treated with cholesteatoma surgery (using the same definition as for the cases). In the main analysis, cases and controls were considered exposed if at least 1 first-degree relative had been treated with cholesteatoma surgery before or after the index date. Cases and controls were considered unexposed if at least 1 first-degree relative was identified but had no record of cholesteatoma in the National Patient Register. Additionally, the exposure was defined by relationship (ie, having a parent [mother and father separately], sibling, and child) with cholesteatoma.

**Figure.**
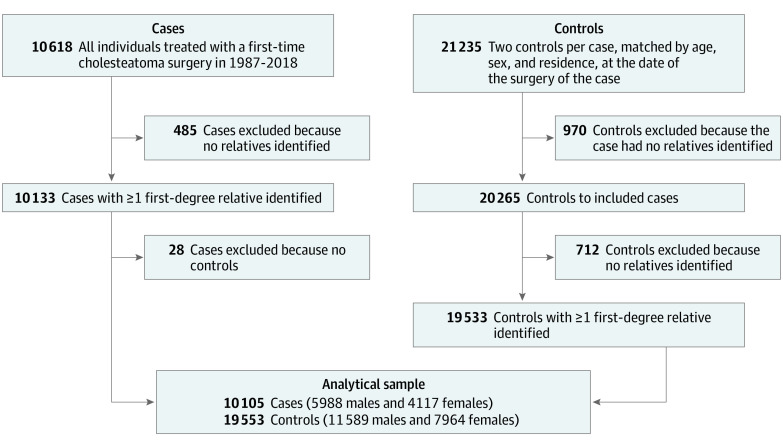
Flowchart of Included Study Population and Analytical Sample

To elucidate the role of increased awareness of the condition (eg, if a close family member had been treated for cholesteatoma it might be more likely that the condition is diagnosed and treated also in the index person), the exposure was defined as having a partner that had been treated with cholesteatoma surgery (before or after the index date). Partners to cases and controls were identified from the Total Population Register in the year preceding the index date, or within the same year. Partners were defined as marital partners for the full study period, and additionally as cohabitants with common children (from 1990 and onwards) or cohabitants without common children (from 2011 and onwards).

### Statistical Analysis

Baseline characteristics were compared between cases and controls using Fisher exact test, and a *P* value ≤.05 was considered statistically significant. The association between having a first-degree relative with cholesteatoma and the risk of cholesteatoma surgery in the index persons were estimated by calculating odds ratios (ORs) and 95% CIs using conditional logistic regression. These analyses are inherently adjusted for the matching factors age, sex, and region of residence. As the controls are drawn at the time of surgery (ie, by incidence density sampling), the ORs can be interpreted as incidence rate ratios.^15^

The main analysis assessed the risk of cholesteatoma surgery by having any first-degree relative with cholesteatoma (yes or no), and analyses were also performed separately by relationship. Since familial cholesteatoma may arise earlier in life, the analyses were stratified by age at index date (<20 years old or ≥20 years old). The diagnosis of cholesteatoma could only be assessed from 1987 and onwards, among both index persons and relatives, and to assess the potential effect of this left truncation of the data, the analyses were stratified by index period (1987-1999 or 2000-2018). Finally, the analyses were stratified by cholesteatoma surgery extension (atticus or mastoid vs other). The estimates of the stratified analyses were compared using the method described by Altman and Bland.^16^ To assess the potential effect of increased health awareness, the risk of cholesteatoma was compared between individuals with and without a partner with cholesteatoma.

All analyses were restricted to cases and controls who had at least 1 first-degree relative identified, that were registered in Sweden any time between 1987 and 2018, in the category of interest (eg, mother, father, child, sibling). Data were received in April 2022, and analyses were conducted between April and September 2022. All analyses were conducted in IBM SPSS Statistics for Windows, version 28.0.0.0 (IBM) or SAS statistical software, version 9.4 (SAS Institute Inc).

## Results

Between 1987 and 2018, 10 618 individuals with a first-time cholesteatoma surgery were identified in the Swedish National Patient Register. Men accounted for 6302 (59.4%) individuals, and mean (SD) age at surgery was 35.6 (21.5) years (34.3 [21.2] for men and 37.4 [21.7] for women, Table 1). Most cases of cholesteatoma were found between the ages of 8 and 18 years old (eFigure 1 in the Supplement). In addition to the cases, 21 235 matched controls were included. The mortality in cases and controls were similar; 1472 cases (13.9%) and 2791 controls (13.1%) died during the study period.

**Table 1.** Descriptive Characteristics of Patients Treated With Surgery for Cholesteatoma During 1987-2018 in Sweden and Matched Controls From the General Population

Characteristic	No. (%)^a^		*P* value^b^
	Cases	Controls	
Total	10 618	21 235	NA
Age at index date, mean (SD)	35.6 (21.5)	35.6 (21.5)	NA
Sex			
Male	6302 (59.4)	12 603 (59.4)	NA
Female	4316 (40.6)	8632 (40.6)	NA
Index year			
1987-1999	4770 (44.9)	9540 (44.9)	NA
2000-2018	5848 (55.1)	11 695 (55.1)	NA
Born in Sweden	8786 (82.7)	18 633 (87.7)	<.001
First-degree relatives			
No. of relatives identified, mean (SD)^c^	4.6 (2.6)	4.6 (2.4)	NA
At least 1 first-degree relative identified	10 133 (95.4)	20 412 (96.1)	.004
Mother identified	7556 (71.2)	16 147 (76.0)	<.001
Father identified	6820 (64.2)	14 571 (68.6)	<.001
Sibling(s) identified	7725 (72.8)	16 083 (75.7)	<.001
Child(ren) identified	6810 (64.1)	13 586 (64.0)	.78
At least 1 male first-degree relative identified	9539 (89.8)	19 250 (90.7)	.02
At least 1 female first-degree relative identified	9606 (90.5)	19 449 (91.6)	.001
Partners			
Having a registered partner	4257 (40.1)	8291 (39.0)	.07

Abbreviation: NA, not applicable.

^a^ The numbers presented for first-degree relatives, siblings, and children are cases and controls with at least 1 relative/sibling/child with cholesteatoma. Ten cases had 2 first-degree relatives with cholesteatoma.

^b^ *P* values are calculated with Fisher exact test.

^c^ Refers to relatives that have been registered in Sweden any time between 1987 and 2018. One individual may have first-degree relationships to several index persons and then be included more than 1 time.

In total, 146 202 first-degree relatives to cases and controls were identified (that were registered in Sweden at any time between 1987 and 2018), with a mean value of 4.6 relatives per index person (for both cases and controls). For 10 133 cases (95.4%) and 20 412 controls (96.1%), at least 1 first-degree relative were identified (Table 1).

Among the 10 105 cases with at least 1 control included in the main analysis (Figure), 227 [2.2%] had at least 1 first-degree relative treated for cholesteatoma, while the corresponding numbers for controls were 118 of 19 553 control patients (0.6%, Table 2). The risk of cholesteatoma surgery among individuals with a first-degree relative treated for the disease, compared with individuals with no affected first-degree relatives was estimated at an OR of 3.9 (95% CI, 3.1-4.8) (Table 2, eFigure 2 in the Supplement). The association differed slightly by sex of the index person, and the OR was higher among men (OR, 4.2; 95% CI, 3.1-5.6) than women (OR, 3.4; 95% CI, 2.4-4.9). The increased risk of cholesteatoma surgery among individuals with affected first-degree relatives were seen for all relationships, with effect sizes spanning between 2.9 (95% CI, 1.9-4.6) for having a child treated for cholesteatoma to 4.4 (95% CI, 3.2-6.2) for having a sibling treated for the disease.

**Table 2.** Odds Ratios of Cholesteatoma Associated With Having at Least 1 First-degree Relative Surgically Treated for Cholesteatoma, in Total and by Type of Relative^a^

Type of relative with cholesteatoma	All			Male patients			Female patients		
	Exposed, No. (%)		OR (95% CI)	Exposed, No. (%)		OR (95% CI)	Exposed, No. (%)		OR (95% CI)
	Cases	Controls		Cases	Controls		Cases	Controls	
Any first-degree relative	227 (2.2)	118 (0.6)	3.9 (3.1-4.8)	138 (2.3)	65 (0.6)	4.2 (3.1-5.6)	89 (2.2)	53 (0.7)	3.4 (2.4-4.9)
Male first-degree relative	139 (1.5)	60 (0.3)	4.5 (3.3-6.1)	83 (1.5)	34 (0.3)	4.6 (3.1-6.9)	56 (1.5)	26 (0.4)	4.3 (2.7-6.8)
Female first-degree relative	90 (1.0)	52 (0.3)	3.4 (2.4-4.8)	56 (1.0)	27 (0.3)	4.0 (2.5-6.3)	34 (0.9)	25 (0.4)	2.7 (1.6-4.6)
Mother	28 (0.4)	17 (0.1)	3.1 (1.7-5.7)	18 (0.4)	10 (0.1)	3.4 (1.6-7.5)	10 (0.4)	7 (0.1)	2.7 (1.0-7.0)
Father	27 (0.4)	12 (0.1)	4.2 (2.1-8.4)	19 (0.5)	10 (0.1)	3.6 (1.7-7.7)	8 (0.3)	<5	7.6 (1.6-35.7)
Sibling(s)	119 (1.6)	48 (0.4)	4.4 (3.2-6.2)	77 (1.7)	26 (0.3)	5.3 (3.4-8.3)	42 (1.5)	22 (0.4)	3.4 (2.0-5.8)
Child(ren)	51 (0.8)	31 (0.3)	2.9 (1.9-4.6)	24 (0.7)	15 (0.3)	2.8 (1.5-5.4)	27 (1.0)	16 (0.3)	3.0 (1.6-5.7)

Abbreviation: OR, odds ratio.

^a^ The analyzed population includes only cases and (their corresponding) controls where a relative could be identified, therefore the total number differs compared with Table 1.

Table 3 presents the analysis stratified by age, type of surgery, and time period of surgery. The OR was higher for individuals with cholesteatoma surgery performed under the age of 20 years (OR, 5.2; 95% CI, 3.5-7.5) as compared with individuals aged 20 years or older (OR, 3.2; 95% CI, 2.4-4.3), and for cases where surgery involved the atticus and/or mastoid areas (OR, 4.9; 95% CI, 3.6-6.6) compared with other locations (OR, 2.9; 95% CI, 2.1-4.0). In contrast, the differences in the associations between earlier (1987-1999; OR, 4.2; 95% CI, 2.9-5.9), and later (2000-2018; OR, 3.7; 95% CI, 2.7-4.9) time periods were much smaller.

**Table 3.** Odds Ratios of Cholesteatoma Associated With Having at Least 1 First-degree Relative Surgically Treated for Cholesteatoma, Stratified by Time Period, Age, and Type of Surgery

Characteristic	All			Male patients			Female patients		
	Exposed, No. (%)		OR (95% CI)	Exposed, No. (%)		OR (95% CI)	Exposed, No. (%)		OR (95% CI)
	Cases	Controls		Cases	Controls		Cases	Controls	
**Time period of surgery**									
1987-1999	98 (2.2)	47 (0.5)	4.2 (2.9-5.9)	62 (2.3)	29 (0.6)	4.2 (2.7-6.6)	36 (2.0)	18 (0.5)	4.1 (2.3-7.2)
2000-2018	129 (2.3)	71 (0.7)	3.7 (2.7-4.9)	76 (2.3)	36 (0.6)	4.2 (2.8-6.2)	53 (2.3)	35 (0.8)	3.1 (2.0-4.8)
*P* value for difference^a^	.59			>.99			.45		
**Age at surgery, y**									
<20	97 (2.9)	38 (0.6)	5.2 (3.5-7.5)	71 (3.4)	23 (0.6)	6.1 (3.8-9.8)	26 (2.1)	15 (0.6)	3.6 (1.9-6.9)
≥20	130 (1.9)	80 (0.6)	3.2 (2.4-4.3)	67 (1.7)	42 (0.6)	3.1 (2.1-4.6)	63 (2.2)	38 (0.7)	3.4 (2.2-5.1)
*P* value for difference^a^	.05			.03			.88		
**Type of surgery**									
Atticus or mastoid	141 (2.5)	58 (0.5)	4.9 (3.6-6.6)	88 (2.6)	37 (0.6)	4.7 (3.2-6.9)	53 (2.4)	21 (0.5)	5.1 (3.1-8.6)
Other locations	86 (1.9)	60 (0.7)	2.9 (2.1-4.0)	50 (1.9)	28 (0.6)	3.5 (2.2-5.5)	36 (1.9)	32 (0.9)	2.3 (1.4-3.7)
*P* value for difference^a^	.02			.33			.03		

Abbreviation: OR, odds ratio.

^a^ *P* values are estimated with the Altman and Bland^16^ method.

Approximately 40% of the cases and controls had a partner who could be identified in the registers (Table 1). There was no difference in the proportion of cases and controls that had a partner treated for cholesteatoma (10 cases [0.3%] and 16 controls [0.3%]; OR, 0.92; 95% CI, 0.41-2.05, Table 4).

**Table 4.** Odds Ratios of Cholesteatoma Associated With Having a Partner Surgically Treated for Cholesteatoma Among Cases and Controls^a^

Affected partner	All			Male patients			Female patients		
	Exposed, No. (%)		OR (95% CI)	Exposed, No. (%)		OR (95% CI)	Exposed, No. (%)		OR (95% CI)
	Cases (n =3611)	Controls (n = 5427)		Cases	Controls		Cases	Controls	
Partner with cholesteatoma	10 (0.3)	16 (0.3)	0.92 (0.41-2.05)	4 (0.2)	11 (0.3)	0.5 (0.2-1.7)	6 (0.4)	5 (0.2)	1.8 (0.5-5.9)

Abbreviation: OR, odds ratio.

^a^ The analyzed population includes only cases and (their corresponding) controls where a partner could be identified, therefore the total number differs compared with Table 1.

## Discussion

This population-based case-control study shows an almost 4-fold–increased risk for cholesteatoma in individuals having at least 1 first-degree relative surgically treated for the disease, compared with individuals with no affected relatives. The association was particularly strong in young individuals and those whose surgery involved atticus and/or mastoid areas. No association was found when comparing the likelihood of having a partner treated for cholesteatoma. This strengthens the hypothesis of a hereditary disease rather than an association driven by increased health awareness.

This nationwide, register-based, case-control study in Sweden included a large number of patients and investigated the risk of family clustering of cholesteatoma during a 30-year long period. Utilizing the Swedish health and population registers, all individuals surgically treated for a cholesteatoma were included, independent on surgical unit performing the operation, which minimizes the risk of selection into the study. Moreover, the tax-funded Swedish health care system offers an equal medical coverage for all citizens minimizing a socioeconomically induced selection bias. All first-degree relatives registered in Sweden during this period were identified, and their disease history, assessed through health data registers; thus, there is no misclassification due to self-reporting of exposure.

Some case reports of familiar clustering of cholesteatoma are found in the literature^9,17,18^ indicating a possibility of a hereditary pattern. In 2014, Djurhuus et al^19^ found a 2-fold risk for cholesteatoma surgery in siblings to individuals with cleft palate but no difference in the groups with lip or combined lip and palate cleft, compared with a random sample of the Danish population. Such distinction cannot be made in the current study’s data because no information of malformations was available.

Having a first-degree relative with cholesteatoma increased the risk of a first cholesteatoma surgery particularly in the younger ages (<20 years at surgery). This could imply that inheritance is of higher importance for childhood cholesteatomas. A few case studies have been published regarding familial clustering of congenital cholesteatomas.^20,21,22,23^ However, the register data used in the current study could not distinguish between congenital and acquired cholesteatoma due to the lack of unique ICD codes, making this impossible to investigate without additional information from medical records. Yet, a recent meta-analysis^24^ regarding congenital cholesteatoma stated that the mean age at surgery was 4.9 years, and in the current study, the most common age at cholesteatoma surgery was 10 years (eFigure 1 in the Supplement). The number of surgeries in patients younger than 7 years in Sweden is very low, accounting for 6.8% of all surgeries (eFigure 1 in the Supplement). The incidence of congenital cholesteatoma is calculated to be between approximately 4% to 24% of all cholesteatomas and the incidence has been shown to be increasing over the last years in South Korea.^25,26^ This said, the influence of a possible inheritance in congenital cholesteatomas affecting the current study’s results cannot be excluded.

The results of the current study indicated slightly more pronounced associations among men compared with women, although the differences were not statistically significant. Male patients have a higher incidence of both otitis media and cholesteatoma in the population,^1,2,3,27^ but the results in this study also point toward a higher susceptibility to inheritance. Several studies have shown a strong genetic component for otitis media in general,^28,29^ and a high percentage of individuals surgically treated for cholesteatoma has a history of otitis media. Due to limitations in register data, the frequency of childhood otitis media was not possible to investigate. The heredity seen in this study could therefore be explained, completely or partly, by the heredity for otitis media and thus have similar origin.

In 2 large cohort studies, the incidence of cholesteatoma was increased in children treated with ventilation tube insertion for otitis media,^30,31^ and several studies have shown a higher incidence in individuals with craniofacial anomalies, such as cleft palate, Turner syndrome, and Down syndrome.^19,32,33^ Except for a higher incidence of cholesteatoma in siblings to individuals with cleft palate,^19^ no studies have been performed for the other subgroups in order to investigate their part in the pattern of inheritance. Further studies in this area are needed.

Having a first-degree relative with cholesteatoma was accompanied with a particularly increased risk for cholesteatoma surgery involving the atticus or mastoid region. This may indicate a different mechanism for cholesteatomas originating in the mesotympanic area. More studies combining register and clinical data are needed to further investigate a difference in cholesteatoma type and inheritance pattern.

### Limitations

Despite a strong relative association between having a first-degree relative with cholesteatoma and the risk of the disease, the number of exposed cases was low, 227 of 10 105 cases (2.2%). This implies that the association only explains a limited number of cholesteatoma occurrences. Interpreting the absolute prevalence of family history of cholesteatoma should be done with caution due to a lack of information regarding cholesteatoma surgeries before 1987 in the current study. This means that cholesteatoma surgery among relatives occurring earlier than 1987 are lacking, and thus, some exposed individuals are misclassified as nonexposed giving an underestimation of the absolute prevalence of family history of cholesteatoma. However, this has outcomes for both cases and controls in the same way, and because this is a rare exposure, the potential effect on the relative estimates are at the most only a minor dilution. This left truncation of the data also implied that some of the cases in the early time period might be included in the study at their second instead of at their primary surgery.

Cases were identified using both diagnostic and procedure codes. This strengthens the sensitivity of the case ascertainment compared with using only diagnostic codes. However, this also means that cases not surgically treated or misdiagnosed are not included, which might lead to a minor loss of precision of the current study’s estimates. Using procedure codes and registry data also leads to a possibility of an overdiagnosis if surgeons have used a cholesteatoma diagnosis when performing surgery for reconstruction purposes secondary to a cholesteatoma surgery. However, that would only overestimate the number of cases during the early time period and to some extent identify the false negative (ie, cases treated in the years prior to 1987). However, results from analyses stratified by time periods were largely similar, indicating only a minor potential effect from these potential biases.

## Conclusions

This case-control study shows an almost 4-fold risk estimate for cholesteatoma in individuals with a first-degree relative surgically treated for the disease. This association was not explained by an enhanced awareness of the condition within families, as no association was found with having a partner with the outcome. Although the relative association was strong, family history was nevertheless quite rare and can therefore only explain a limited number of all cholesteatoma cases. However, these families could be an important source for information regarding the genetic background for cholesteatoma disease. The use of nationwide register data with high coverage and completeness strengthens the result. Future studies including second-degree relatives and information from medical records could further illuminate the association of heredity.

## Notes

Supplement 1. **eFigure 1.** Number of first surgeries for cholesteatoma in regard to age at surgery (in years) registered in the Swedish National Patient Register between 1987-2018 for women and men **eFigure 2.** Forest plot showing odds ratio with 95% CIs for first-degree relatives in total, having a male or a female first-degree relative, and all relatives separate **eTable.** Swedish ICD-9 and ICD-10 codes used, in combination with one or several of the Swedish procedural codes, for identifying individuals surgically treated for cholesteatoma

Supplement 2. Data sharing statement
