# Supplementary material for: The Risk of Cholesteatoma in Individuals With First-degree Relatives Surgically Treated for the Disease
Source: JAMA Otolaryngol Head Neck Surg. 2023 Mar 16;149(5):390–6. doi: 10.1001/jamaoto.2023.0048 (PMC10020932; doi:10.1001/jamaoto.2023.0048)
Supplement: Supplement 1. — eFigure 1. Number of first surgeries for cholesteatoma in regard to age at surgery (in years) registered in the Swedish National Patient Register between 1987-2018 for women and men eFigure 2. Forest plot showing odds ratio with 95% CIs for first-degree relatives in total, having a male or a female first-degree relative, and all relatives separate eTable. Swedish ICD-9 and ICD-10 codes used, in combination with one or several of the Swedish procedural codes, for identifying individuals surgically treated for cholesteatoma [file jamaotolaryngolheadnecksurg-e230048-s001.pdf]

Supplemental Online Content

Bonnard Å, Berglin CE, Wincent J, et al. The risk of cholesteatoma in individuals with first-degree relatives surgically treated for the disease. *JAMA Otolaryngol Head Neck Surg.* Published online March 16, 2023. doi:10.1001/jamaoto.2023.0048

eFigure 1. Number of first surgeries for cholesteatoma in regard to age at surgery (in years) registered in the Swedish National Patient Register between 1987-2018 for women and men

eFigure 2. Forest plot showing odds ratio with 95% CIs for first-degree relatives in total, having a male or a female first-degree relative, and all relatives separate

eTable. Swedish *ICD-9* and *ICD-10* codes used, in combination with one or several of the Swedish procedural codes, for identifying individuals surgically treated for cholesteatoma

This supplemental material has been provided by the authors to give readers additional information about their work.

© 2023 Bonnard Å et al. *JAMA Otolaryngology–Head & Neck Surgery.*

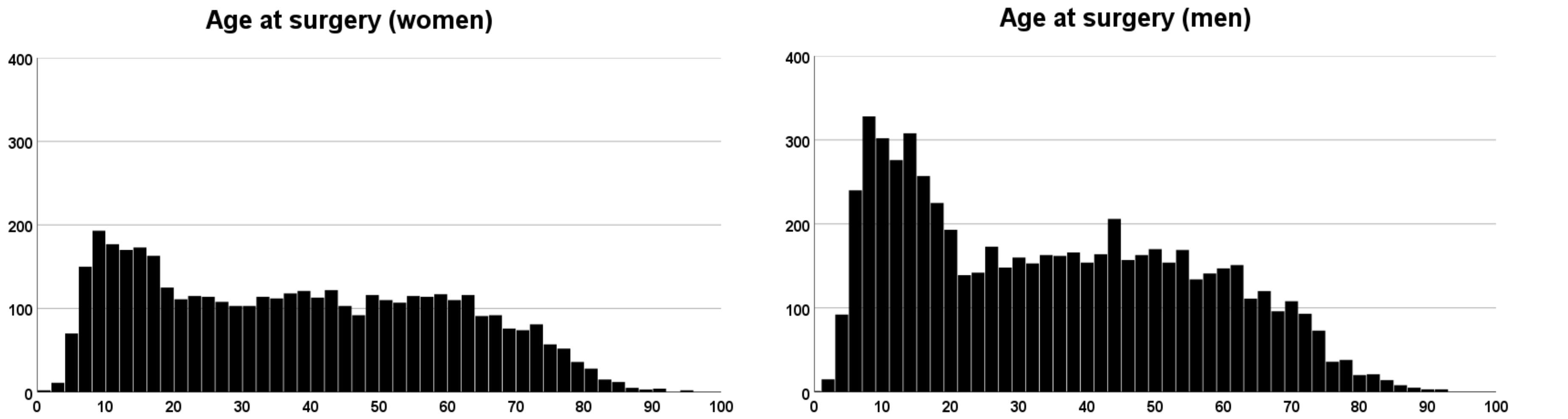

eFigure 1. Number of first surgeries for cholesteatoma in regard to age at surgery (in years) registered in the Swedish National Patient Register between 1987-2018 for women and men. The number of surgeries and the mean age for women was 4 316 and 37.4 years respectively, and for men 6 302 surgeries and 34.3 years.

© 2023 Bonnard Å et al. *JAMA Otolaryngology–Head & Neck Surgery.*

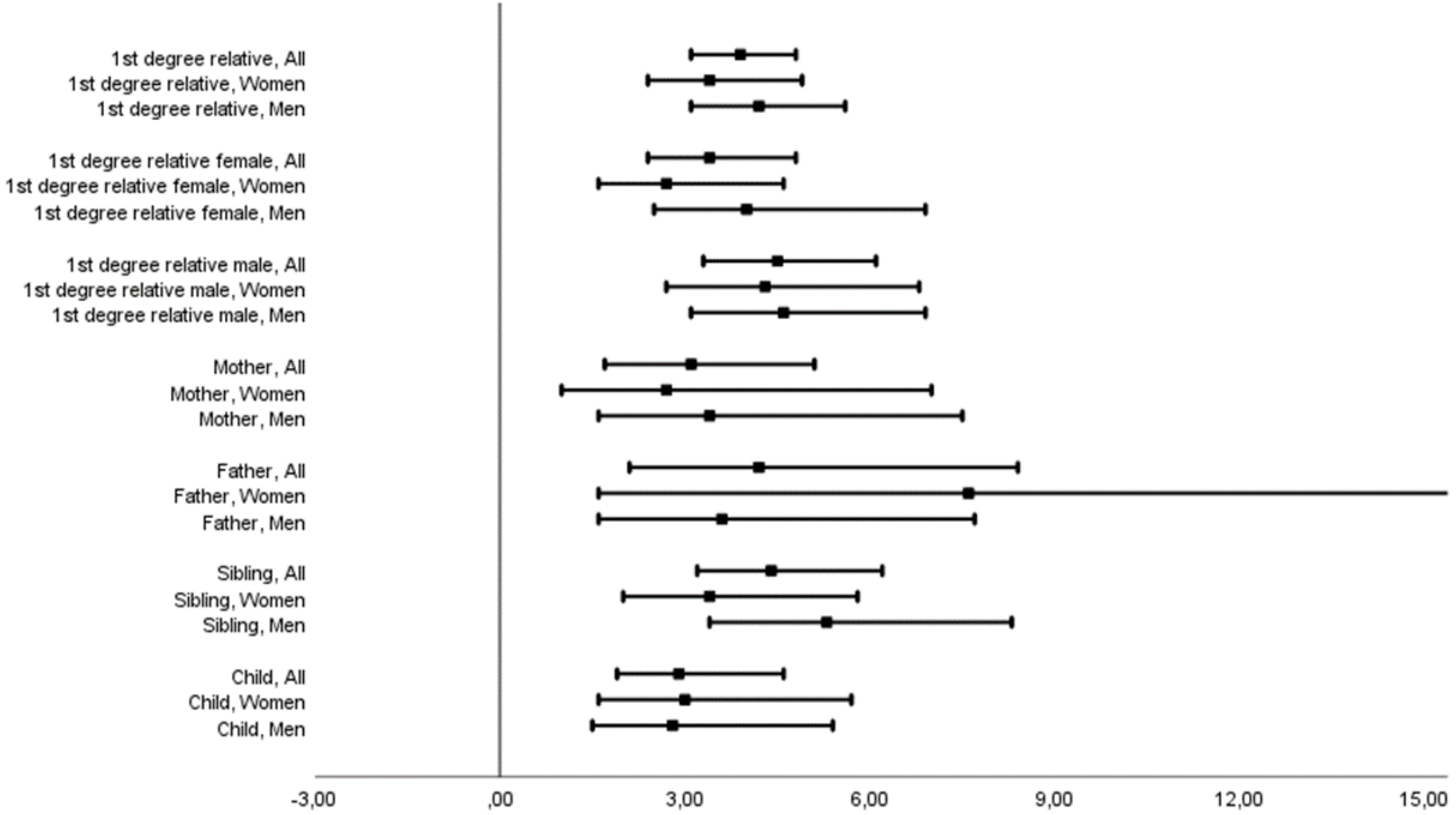

eFigure 2. Forest plot showing odds ratio with 95% CIs for first-degree relatives in total, having a male or a female first-degree relative, and all relatives separate. Each result is divided in three parts, all in total and for women and men separately.

© 2023 Bonnard Å et al. *JAMA Otolaryngology–Head & Neck Surgery.*

Code for Diagnosis:

Classification	ICD-9	ICD-10
	**385D (385.3 Cholesteatoma of middle ear and mastoid)**	**H71.9 (H71 Cholesteatoma of middle ear)**
	383D (383.3 Complications following mastoidectomy)	H95.0 (Recurrent cholesteatoma of postmastoidectomy cavity)

Swedish Procedural Terminology codes:

Year	1963-96	1997-
	2042 (Myringoplasty)	DCA30 (Exploration of the middle ear)
	2052 (Tympanotomy)	DCB00 (Polyp extraction)
	2056 (Ossiculoplasty)	DCD00 (Myringoplasty)
	2059 (Other surgeries on the ossicles)	DCD10 (Tympanoplasty)
	**2070-78 (Different mastoidectomies)**	DCW99 (Other surgery on ear drum or middle ear)
	**2080 (Atticoantrotomy)**	DDA10 (Exploration of ossicular chain)
	2081-88 (Posterior tymanoctomy, total reconstruction, other similar surgeries)	DDD05/10/20 (Different ossicular plasties)
		**DEA10 (Endural atticoantrotomy)**
		**DEB00/10/20/25/30/40 (Different mastoidectomies)**
		DED00 (Obliteration of radical cavity)

eTable. Swedish *ICD-9* and *ICD-10* codes used, in combination with one or several of the Swedish procedural codes, for identifying individuals surgically treated for cholesteatoma. Disease and procedural codes in bold letters were used in the distinction between surgeries involving the attic and/or mastoid areas compared to the other locations.

© 2023 Bonnard Å et al. *JAMA Otolaryngology–Head & Neck Surgery.*
