# Supplementary material for: The Risk of Cholesteatoma in Individuals With First-degree Relatives Surgically Treated for the Disease
Source: JAMA Otolaryngol Head Neck Surg. 2023 Mar 16;149(5):390–6. doi: 10.1001/jamaoto.2023.0048 (PMC10020932; doi:10.1001/jamaoto.2023.0048)
Supplement: Supplement 2. — Data sharing statement [file jamaotolaryngolheadnecksurg-e230048-s002.pdf]

Data Sharing Statement

Bonnard. The Risk of Cholesteatoma in Individuals With First-degree Relatives Surgically Treated for the Disease. *JAMA Otolaryngol Head Neck Surg*. Published March 16, 2023. doi:10.1001/jamaoto.2023.0048

Data
Data available: Yes
Data types: Other (please specify)
Additional Information: "These data are available for research purposes through applications to each register holder after ethical review and secrecy assessment."
How to access data: Data is available from Statistics Sweden (https://www.scb.se/vara-tjanster/bestalla-mikrodata/), the Swedish National Board of Health and Welfare (https://bestalladata.socialstyrelsen.se/data-for[1]forskning/ after ethical approval
When available: beginning date: 01-01-2022

Supporting Documents
Document types: None

Additional Information
Who can access the data: All researchers whose proposed use of the data has been approved after ethical approval and application to the above mentioned registers
Types of analyses: All purposes included by the approved ethical permission
Mechanisms of data availability: without investigator support and after above mentioned ethical approval
